# Supplementary material for: Characterisation of tumour microenvironment remodelling following oncogene inhibition in preclinical studies with imaging mass cytometry
Source: Nat Commun. 2021 Oct 8;12:5906. doi: 10.1038/s41467-021-26214-x (PMC8501076; doi:10.1038/s41467-021-26214-x)
Supplement: Supplementary file 3 — Reporting Summary [file 41467_2021_26214_MOESM3_ESM.pdf]

nature research

Corresponding author(s): Julian Downward, Febe van Maldegem

Last updated by author(s): Sep 5, 2021

Reporting Summary

Nature Research wishes to improve the reproducibility of the work that we publish. This form provides structure for consistency and transparency in reporting. For further information on Nature Research policies, see our Editorial Policies and the Editorial Policy Checklist.

Statistics

For all statistical analyses, confirm that the following items are present in the figure legend, table legend, main text, or Methods section.

n/a	Confirmed	
☐	☒	The exact sample size (*n*) for each experimental group/condition, given as a discrete number and unit of measurement
☐	☒	A statement on whether measurements were taken from distinct samples or whether the same sample was measured repeatedly
☐	☒	The statistical test(s) used AND whether they are one- or two-sided<br>*Only common tests should be described solely by name; describe more complex techniques in the Methods section.*
☐	☒	A description of all covariates tested
☐	☒	A description of any assumptions or corrections, such as tests of normality and adjustment for multiple comparisons
☐	☒	A full description of the statistical parameters including central tendency (e.g. means) or other basic estimates (e.g. regression coefficient) AND variation (e.g. standard deviation) or associated estimates of uncertainty (e.g. confidence intervals)
☐	☒	For null hypothesis testing, the test statistic (e.g. *F*, *t*, *r*) with confidence intervals, effect sizes, degrees of freedom and *P* value noted<br>*Give P values as exact values whenever suitable.*
☒	☐	For Bayesian analysis, information on the choice of priors and Markov chain Monte Carlo settings
☒	☐	For hierarchical and complex designs, identification of the appropriate level for tests and full reporting of outcomes
☒	☐	Estimates of effect sizes (e.g. Cohen's *d*, Pearson's *r*), indicating how they were calculated

Our web collection on statistics for biologists contains articles on many of the points above.

Software and code

Policy information about availability of computer code

Data collection	CyTOF Software v6.7 and v.7.0, Analyze v12.0, Zen blue imaging software (Zeiss)
Data analysis	Segmentation: IMCtools v1.0.5, Ilastik v1.3.3b1, CellProfiler v3.1.9, Imcyto v1.0.0, R v3.6.2, Code availability: https://hdl.handle.net/10779/crick.c.5270621.v1, https://nf-co.re/imcyto, https://github.com/nf-core/imcyto, https://github.com/FrancisCrickInstitute/vanMaldegem_Valand_2021, https://doi.org/10.5281/zenodo.3865430, https://doi.org/10.5281/zenodo.5412513

For manuscripts utilizing custom algorithms or software that are central to the research but not yet described in published literature, software must be made available to editors and reviewers. We strongly encourage code deposition in a community repository (e.g. GitHub). See the Nature Research guidelines for submitting code & software for further information.

Data

Policy information about availability of data

All manuscripts must include a data availability statement. This statement should provide the following information, where applicable:

- Accession codes, unique identifiers, or web links for publicly available datasets
- A list of figures that have associated raw data
- A description of any restrictions on data availability

Data availability: https://hdl.handle.net/10779/crick.c.5270621.v1

Field-specific reporting

Please select the one below that is the best fit for your research. If you are not sure, read the appropriate sections before making your selection.

☒ Life sciences ☐ Behavioural & social sciences ☐ Ecological, evolutionary & environmental sciences

For a reference copy of the document with all sections, see nature.com/documents/nr-reporting-summary-flat.pdf

Life sciences study design

All studies must disclose on these points even when the disclosure is negative.

Sample size	We chose to use an even number of images for both treatment groups, with a variety of tumour sizes. As effect size and variation could not be estimated prior to this experiment, being first in its kind, we based the sample size on experience with flow cytometry experiments where usually 5-6 samples per group is sufficient to perform statistical testing.
Data exclusions	From two images small areas were excluded because of a sudden drop in counts from the mass spec. These are detailed in the rPhenograph_clustering.R script. This relates to the dataset used in figures 3-6 and Supplementary figures S4-S7
Replication	The changes seen in tumour microenvironment, related to cell numbers and phenotypes, have been successfully confirmed with flow cytometry in two independent experiment. The Imaging Mass Cytometry experiment was conducted only once, due to its size, cost and analysis time (2 years)
Randomization	Mice were divided into two groups with equivalent tumour burden before randomly assigning each of the groups to a treatment. No other randomisation applicable.
Blinding	Therapeutic treatment of mice was done in a blinded setup, where the animal technician delivered the drug from coded vials, unaware of the treatment. Immunohistochemical staining and scanning of the tissues was done blinded for treatment history. Data normalisation, clustering and cell type annotation was performed on the dataset as a whole, without considering treatment history.

Reporting for specific materials, systems and methods

We require information from authors about some types of materials, experimental systems and methods used in many studies. Here, indicate whether each material, system or method listed is relevant to your study. If you are not sure if a list item applies to your research, read the appropriate section before selecting a response.

Materials & experimental systems

n/a	Involved in the study	
☐	☒	Antibodies
☐	☒	Eukaryotic cell lines
☒	☐	Palaeontology and archaeology
☐	☒	Animals and other organisms
☒	☐	Human research participants
☒	☐	Clinical data
☒	☐	Dual use research of concern

Methods

n/a	Involved in the study	
☒	☐	ChIP-seq
☒	☐	Flow cytometry
☒	☐	MRI-based neuroimaging

Antibodies

Antibodies used	This information is supplied in Supplementary Tables 1 and 2
Validation	Validation of the antibodies has been described in the main text of the manuscript and shown in Supplementary figure 2

Eukaryotic cell lines

Policy information about cell lines

Cell line source(s)	3LL, derived from Lewis Lung Carcinoma, modified with deletion of the NRAS gene, previously described in Molina-Arcas, et al. Sci. Transl. Med. 2019
Authentication	Cell line has been sequenced by WES and contains largely the same mutations as the cell line LL/2 (LLC1) from ATCC
Mycoplasma contamination	Cell line was regularly tested for Mycoplasma and found negative.
Commonly misidentified lines (See ICLAC register)	No commonly misidentified cell lines were used in the study

Animals and other organisms

Policy information about studies involving animals; ARRIVE guidelines recommended for reporting animal research

Laboratory animals	male C57BL/6 mice, aged 9-11 weeks at tumour inoculation
Wild animals	No wild animals were used in the study.
Field-collected samples	No field-collected samples were used in the study.
Ethics oversight	This work was performed under a UK Home office approved project license and in accordance with institutional (Crick) welfare guidelines.

Note that full information on the approval of the study protocol must also be provided in the manuscript.
